# A transcriptional dynamic network during *Arabidopsis thaliana *pollen development

**DOI:** 10.1186/1752-0509-5-S3-S8

**Published:** 2011-12-23

**Authors:** Jigang Wang, Xiaojie Qiu, Yuhua Li, Youping Deng, Tieliu Shi

**Affiliations:** 1 College of Life Sciences, Northeast Forestry University, Heilongjiang, Harbin 150040, China; 2 The Center for Bioinformatics and The Institute of Biomedical Sciences, School of Life Sciences, East China Normal University, Shanghai 200241, China; 3 Wuhan University of Science and Technology, Wuhan, Hubei 430081, P.R. China; 4 Department of Internal Medicine, Rush University Medical Center, Chicago, Illinois 60612, USA; 5 Shanghai Information Center for Life Sciences, Shanghai Institutes for Biological Sciences, Chinese Academy of Science, Shanghai 200031, China

## Abstract

**Background:**

To understand transcriptional regulatory networks (TRNs), especially the coordinated dynamic regulation between transcription factors (TFs) and their corresponding target genes during development, computational approaches would represent significant advances in the genome-wide expression analysis. The major challenges for the experiments include monitoring the time-specific TFs' activities and identifying the dynamic regulatory relationships between TFs and their target genes, both of which are currently not yet available at the large scale. However, various methods have been proposed to computationally estimate those activities and regulations. During the past decade, significant progresses have been made towards understanding pollen development at each development stage under the molecular level, yet the regulatory mechanisms that control the dynamic pollen development processes remain largely unknown. Here, we adopt Networks Component Analysis (NCA) to identify TF activities over time couse, and infer their regulatory relationships based on the coexpression of TFs and their target genes during pollen development.

**Results:**

We carried out meta-analysis by integrating several sets of gene expression data related to *Arabidopsis thaliana *pollen development (stages range from UNM, BCP, TCP, HP to 0.5 hr pollen tube and 4 hr pollen tube). We constructed a regulatory network, including 19 TFs, 101 target genes and 319 regulatory interactions. The computationally estimated TF activities were well correlated to their coordinated genes' expressions during the development process. We clustered the expression of their target genes in the context of regulatory influences, and inferred new regulatory relationships between those TFs and their target genes, such as transcription factor WRKY34, which was identified that specifically expressed in pollen, and regulated several new target genes. Our finding facilitates the interpretation of the expression patterns with more biological relevancy, since the clusters corresponding to the activity of specific TF or the combination of TFs suggest the coordinated regulation of TFs to their target genes.

**Conclusions:**

Through integrating different resources, we constructed a dynamic regulatory network of *Arabidopsis thaliana *during pollen development with gene coexpression and NCA. The network illustrated the relationships between the TFs' activities and their target genes' expression, as well as the interactions between TFs, which provide new insight into the molecular mechanisms that control the pollen development.

## Background

Genome specifies the gene expression programs that control cells' differentiation through transcriptional regulatory networks, which are characterized as the dynamic interactions between transcription factors and their target genes during development. Transcription factors regulate the expression of their target genes at transcriptional level with spatiotemporal specificity, thus the modification of transcription factor activity can dramatically alter the gene expression profile. The primary challenge to understand the transcriptional regulation network is to measure the activities of the transcription factors at genome-scale, which are not yet practicable. However, computational methods have recently been developed to infer the transcription factor activities and the regulatory relationships between TFs and their target-genes.

Recent development of high-throughput technologies has made it possible to measure the expression activities of transcription factors and their target genes at the genome-scale. Microarrays can detect the expression levels of thousands of genes simultaneously [1]. But identifying transcription factor activities at such scale is still a challenge, especially for plants. Several technologies for assessing transcriptional activities, such as ChIP-chip, flow cytometer, have their inherent limitation on genome-scale [2-4] and merely detect the activities at specific time point. In order to utilize the genome expression profile and compensate the inability to assay transcription factor activity on the genome-scale, many computational tools have been developed to accomplish this task through inferring gene regulatory networks [5-8]. One of these approaches, Network Component Analysis (NCA) is to determine both activities and regulatory influences for a set of transcription factors on known target genes [9]. It has been successfully applied in several species and in various research perspectives, including yeast cell cycle [9] and cytokinesis-related gene regulation [10], time course of E. coli protein [11], knockout analysis in mouse [12], and transcriptional regulatory network of human [13].

In flowering plants, the male gametophyte (or pollen grain) plays a vital role in plant fertility through generation and delivery of the male gametes to the embryo sac for double fertilization. The male gametophyte development is a complex process that requires the coordinated participation of various cells and tissue types, and their associated specific gene expression patterns. The availability of the genome sequence of *Arabidopsis *(The *Arabidopsis *Genome Initiative, 2000) and the concomitant accumulation in available transcriptional profile data (TAIR) make *Arabidopsis *a preferable model plant for large scale genetic studies of pollen development. In previous studies, several sets of gene expression profiles for *Arabidopsis *pollen development time series have been generated [14-18]. These data cover almost all the stages of *Arabidopsis *pollen development: from uninucleate microspores, bicellular pollen, tricellular pollen, mature pollen grain, the 0.5 hr pollen tube, to 4 hr pollen tube. Besides the availability of those gene expression profile data, the researches on the TFs in Arabidopsis become increasing intensive, and a number of new transcription factors has been identified, either experimentally confirmed or computationally predicted. The total transcription factors of *A. thaliana *are proposed to be more than 2000 according to the four representative databases of *Arabidopsis *transcription factors: RARTF [19], AGRIS [20], DATF [21], PlnTFDB [22]. Among them, a few families of transcription factors have been intensively examined for their functionalities in development. However, the data for regulatory relationships between these transcription factors and their confirmed target genes are very limited.

During the past decade, major advances in genetic and genomic technologies have facilitated our understanding of pollen development at the molecular level. The achievement includes the highly annotated *A. thaliana *genome, comprehensive *A. thaliana *transcriptomic datasets, and various gametophytic mutants. Although significant progress has been made towards understanding pollen development at each development stage, yet the dynamic regulatory network remains further characterized, the transcription factors and their target genes involved in the dynamic processes need investigation in deeper.

By taking advantage of NCA, we explored the regulatory relationships between those TFs and their target genes specifically involved in the *A. thaliana *pollen development process. We identified new regulatory relationships with our most comprehensive dynamic regulatory networks, which provide new information to uncover the underlying mechanisms for the pollen development.

## Results and discussion

When predicting interactions between TFs and their target genes based on gene expression profile, a key assumption is that mRNA expression level is informative in the prediction of protein activity. Although expression levels between mRNAs and their corresponding proteins in different cell types exhibit a range of correlations for different genes [23], an overall positive correlation between mRNA and protein expression levels has been identified [24,25], therefore, we adopt this strategy in our study.

The NCA requires two inputs: a time series of gene expression profiles and a pre-defined regulatory network. The original gene expression data are obtained from the *Arabidopsis *Information Resource (TAIR) and Gene Expression Omnibus (GEO) of NCBI. They cover seven *A. thaliana *pollen developmental stages with 23 profiles in total for wild type Columbia (Col-0): uninucleate microspores (UM), bicellular pollen (BP), tricellular pollen (TP), mature pollen (MP), hydrated pollen grains (HP), 0.5 hours germinated pollen tubes (0.5 hr), and 4 hours germinated pollen tubes (4 hr). Those datasets of pollen developmental stages were generated by three labs [14-16], each of which includes at least one MP sample as control. In order to make comparison between datasets from different labs, the MP sample from that lab is used as the control to process the related dataset, and only the fold change values of each gene from each dataset is kept for the future calculation.

The insufficiency of the availability and comparability of *A. thaliana *pollen development expression data limit the power of NCA. To overcome the limitation, besides we take the mature pollen expression data as the control from the same experiment, we also collect the pollen development-related transcription factors from the Database of Arabidopsis Transcription Factors (DATF), The Arabidopsis Gene Regulatory Information Server (AGRIS), and the Plant Transcription Factor Database (PlnTFDB).

In NCA, the pre-defined regulatory network initially accounts for the gene expression response. The regulatory relationships between the transcription factors and their target genes can be collected from published literatures and transcriptional factors related databases [26]. From the three databases mentioned above, we collect 2, 283 transcription factors which can be mapped to microarray probes. We also collect 8 interaction pairs between transcription factors specific for *A. thaliana *pollen development through text-mining. However, the interaction data between transcription factors and their target genes in pollen development is very limited. Therefore, we have not enough prior interactions available for NCA. To overcome this limitation, we use the microarray data to explore the potential regulatory interactions according to the correlation coefficient (r) of each pair of transcription factors and the fold change (FC) of each gene under different conditions. We choose those gene pairs with correlation coefficient |r|>0.9 and the genes with |FC|>1.6. To reduce false positive data, all differentially expressed genes (DEGs) are hierarchically clustered by FC values, and those genes with high correlation are grouped into corresponding clusters. The resulting clusters indicate that all the genes under a cluster can be regulated by the related TF. Taking the correlation coefficient as control strength for NCA, we define a matrix of regulatory relationships between the selected TFs and their target genes, and generate a regulatory network for the pollen development. The regulatory network includes 289 transcription factors, 5530 target genes and 429, 790 regulatory relations. Processed by NCA, we obtain 15 TFs and 101 target genes. Because of the inability of NCA to predict the regulatory pattern of transcription factors, we take the positive correlation between TF and its target genes as positive regulation, and negative correlation as negative regulatory relation. Based on the network and the expression data, we further estimate the activities of the transcription factors in the network over pollen development with NCA and characterize the dynamic regulatory network. NCA decomposes the matrix of gene expression values into two matrixes, one matrix represents the influence of a transcription factor on a target gene and another reflects the activities of transcription factor [9].

### Transcription factor activities under different pollen development stages

**Figure 1:**
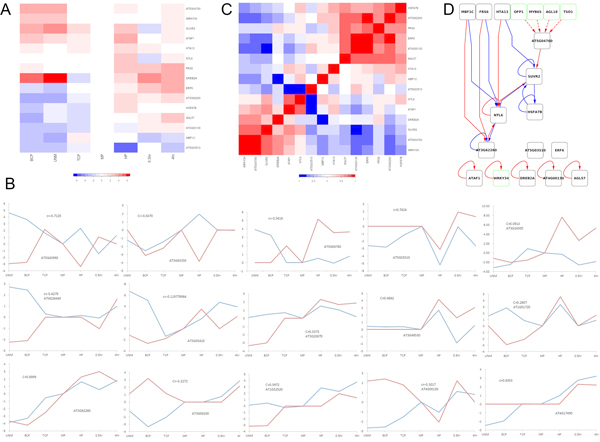
**Transcription factor activities calculated using NCA**. (A) Predicted activities of the fifteen transcription factors used in this study. For each transcription factor, rows represent development stage. Activities of each row are normalized to the MP stage. (B) Transcription factor activities (red) compared to gene expression (blue), with Pearson correlation coefficients noted. Both activity and expression at each time point are normalized to the MP stage values, and the activity is further scaled for direct comparison with the expression values. (C) Correlation matrix between transcription factor activities. Red represents positive correlation, and blue represents negative correlation. (D) Inferred combinatorial regulation pairs of transcription factors. Red lines represent positive regulation, and blue lines represent negative regulation. Green square represents TFs associated with pollen development found by text-mining (The regulation of these TFs are putative).

The activities of 15 TFs clearly show stage-specific actions in pollen and pollen tube development. 12 of them (AT4G17490, AT5G43990, AT5G05410, AT5G04760, AT3G49530, AT5G03510, AT3G63360, AT4G26440, AT3G20670, AT3G24500, AT1G01720, AT1G52520) are activated during pollen development, while the genes for the rest 3 TFs (AT3G63350, AT4G00130, AT3G04100) remain relatively high expression without significant change (Figure 1). AT4G17490 (ATERF6) gene, encoding the ethylene responsive element binding factor 6 [22], belongs to AP2-EREBP gene family and shows its maximum activity at 0.5 hr with a slight decrease at 4 hr in pollen tube development. Previous research has indicated that members in AP2-EREBP gene family play a role in floral organ identity determination [27]. AT5G43990 (SUVR2) gene, its product acting as a histone-lysine N-methyltransferase/zinc ion binding factor [22], is expressed during the fourth anthesis [28], reaching its peak expression at TCP stage and returning to baseline at 4 hr stage during pollen development. SUVR2 is one of SUVR family protein, which can act in concert to achieve various functional H3K9 methylation states that will eventually lead to DNA methylation in a locus-specific manner (Mutskov and Felsenfeld 2004). The up-regulation of SUVR gene in the specific stage of pollen development indicates the involvement of histone remodification in the gene expression switch and regulation rewiring at the epigenetic level during the process. Gene AT5G05410 (DREB2A) is expressed in pollen tube cell, and its activity steadily increased from BCP to HP. DREB2A is an important transcription factor that has been confirmed to involve in heat or water stress-inducible gene expression of *A. thaliana*. It specifically interacts with cis-acting dehydration-responsive element/C-repeat (DRE/CRT), thus functions in cold and drought stress-responsive gene expression in *A. thaliana *[29]. The expression pattern of DREB2A gene indicates that some cold and drought stress related biological processes are also involved in the pollen tube cell development and growth. AT5G04760 (MUK11.7) expression is detected in germinated pollen grain and pollen tube cell, and exhibits a sharp increase from MP to HP stage. AT5G03510 (F12E4.290), a C2H2-type zinc finger family protein, changes its gene expression from HP stage. As a member of heat stress transcription factor family, AT3G63350 (HSFA7B) has been shown to be expressed during the fourth anthesis stage [28], and down-regulated at BCP, HP stage and eventually return to its base level. AT3G62260 gene (T17J13.220, encoding a protein phosphatase 2c family protein), which expression has been reported during the fourth anthesis stage as AT3G63350 does [28], is turned on at TCP stage. AT3G49530 (NTL6), auto-stimulated in pollen tube cell development [30], is up-regulated at HP stage. AT4G26440 (ATWRKY34, a member of WRKY transcription factor family), which gene expression has been detected in anther and pollen tube cell [28], is activated at BCP. Its gene expression has been confirmed as pollen specific [31-33]. AT4G00130 (F6N15.6) gene presents a rapidly reduced activity from BCP to HP and a sharp increase from HP to 0.5 hr stage. AT3G20670 (HTA13) gene, which is expressed in pollen tube cell, increases its expression steadily from UNM to HP stage. AT3G24500 (MBF1C) is a key regulator of a coordinated heat stress-response network involving SA-, trehalose- and ethylene-signaling pathways, and its gene is expressed in pollen tube; its expression is steadily increased from BCP and reaches its peak at HP stage. AT3G04100 (ATAF1) belongs to a large family of putative transcriptional activators with NAC domain; its expression is detected in pollen tube cell and deactivated from BCP stage. As the same family as AT3G04100 with NAC domain, AT1G01720 (ATAF1) gene also shows its expression in pollen tube cell, it is steadily up-regulated from BCM and reaches its peak expression at HP stage. ATAF1 has been proposed to modulate plant ABA signaling and high ATAF1 expression has been considered to contribute to ABA hypersensitivity in Arabidopsis [34]. AT1G52520 (FRS6), which potentially acts as positive regulators in phyB signaling pathway controlling flowering time [35], is steadily up-regulated from UNM and reaches its peak expression at 0.5 hr stage.

The correlations between gene expressions for transcription factors and their activities are not identical among all the transcription factors. Five transcription factors (AT4G17490, AT5G03510, AT3G62260, AT1G52520, AT3G20670) present strong positive correlation between their activities and expressions (r > 0.5), when three transcription factors (AT5G43990, AT5G04760, AT4G26440) show strong negative correlation (r < -0.5). However, the rest seven TFs (AT3G63350, AT5G05410, AT3G49530, AT3G24500, AT3G04100, AT1G01720, and AT4G00130) display less consistence or no correlation at all (|r|< 0.5).

Since the linear model of gene expression upon which NCA rests does not reveal the relationships between transcription factors, we search all the transcription factor pairs with high correlation (|r|> 0.5) from the protein-protein interactions catalogued in the *A. thaliana *Protein Interactome Database [36]. However, no protein-protein interaction has been recorded for any pair of the 15 TFs. Although no experimental data confirms the direct interactions between those TFs, the high correlations between some TFs under different development states suggest their possible relations. Interestingly, the correlation matrix between transcription factor activities reveals that two sets of TFs' activities are apparently positively correlated. One set includes 6 TFs: HSFA7B, AT3G62260, FRS6, ERF6, AT4G00130, and AGL57, another includes WRKY34, AT3G04760, SUVR2. Although no experimental data supports that the TFs in each set form direct interaction, the results inferred from NCA represent an indirect evidence of the interaction or cooperation among them.

### Regulatory influence matrix and gene expression clustering

**Figure 2:**
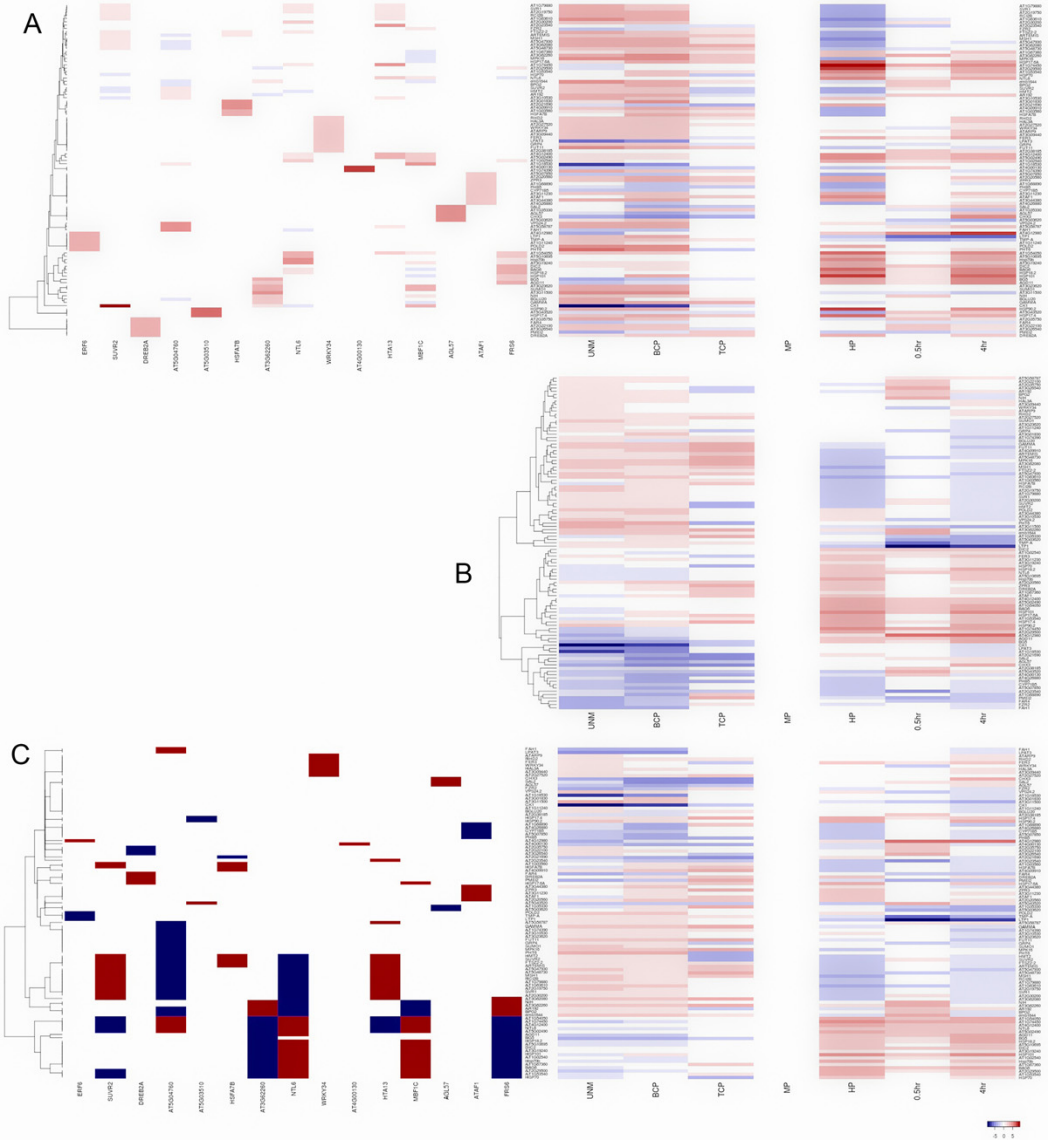
**Hierarchical clustering in the context of a defined regulatory network**. (A) The adjusted strength matrix is used for clustering, with gene expression matrix appended. In the adjusted strength matrix heatmap, red color indicates positive regulatory influence, while blue color indicates negative regulatory influence. (B) Clustering with gene expression only. (C) Clustering with the binary regulatory relations (initial connectivity matrix), assuming the absolute values of all regulatory strengths are equal.

According to the assumptions of NCA, the target gene expression is controlled by an adjusted strength matrix and the transcription factor activities. The assigned quantitative values of the adjusted strength are able to be used to obtain more biologically meaningful clusters than by using target genes' expression. Based on their expressions, the target genes are hierarchically clustered with the adjusted strengths of transcription factors (Figure 2A). In total, eleven major clusters are identified (Additional file 1), which represents the coordinated actions of transcription factors to regulate the gene expression. Cluster 4, 7, 8, and 9 highlight the influence of single TF on a set of genes, whereas cluster 3, 11, 10, and 5 display a set of TFs influence on a set of genes. Interestingly, the regulatory relationships from the clusters can also disclose the auto-regulation of the transcription factors. For example, in the cluster 4, it reveals that the gene AT3G04100 (AGL57), which encodes a MADS-box family protein, is also a target of its own protein, and the same as AT1G01720 in cluster 8, AT1G01720 in cluster 9 and AT4G00130 in cluster 12, as well as AT5G43990, AT5G04760, AT3G63350, AT3G49530 in cluster 3. Those self-regulations are unable to be identified from the coexpression approach. NCA shows certain advantages and the auto-regulation can be inferred from clustering on the matrix of regulation influence.

On the other hand, clustering by regulatory strength can identify new clusters unobtainable by clustering the expression data alone. For example, cluster 9 and 5 could not be distinguished when clustering is applied to the gene expression data alone (Figure 2B). In contrast, those two groups can be separated with clustering on the regulatory strength matrix, and are linked to the regulatory influence of transcription factor DREB2A, HTA13 and NTL6. For the target genes FZR2 and SVR1, they cannot be grouped together with the clustering method on the gene expression data alone (Figure 2B), but they are grouped into cluster 3 based on regulatory strength and supposedly regulated by transcription factors SUVR2, AT5G04760, HSFA7B, AT3G62260, NTL6, HTA13, MBF1C, and FRS6. Furthermore, the clustering of the NCA-processed strength matrix adjusted from the initial connectivity matrix can group genes with different expression patterns (Figure 2A and 2C).

Our results further demonstrate that the estimated transcriptional regulation strengths have certain advantages over the gene coexpression approaches for exploring the regulatory relationships and can provide a new insight to the regulatory relations of between transcription factors and their target genes.

### Coexpression analysis of the regulatory gene sets

Each pair of TF and its target gene(s) classified by NCA have a high correlation coefficient (|p|>0.9) based on gene expression. Considering that our identified regulatory relationships between each TF and its target genes are derived only from process of pollen development, we further test the robustness of the coexpression under other conditions, such as tissue, abiotic and light conditions. We explore each pair of the TFs and its target gene(s) inferred from NCA in ATTED [37] which is a database of gene coexpression in *Arabidopsis *under a wide variety of experiment conditions, and find 65 coexpression pairs (in total 472 identified pairs) with correlation coefficient larger than 0.4 (|r|> 0.4), including 8 TFs and 35 target genes. Almost a quarter (15/65) of these coexpressions are negative. Since the rest 407 TF and target gene pairs display the low correlation under all other experimental conditions but show a high correlation in pollen development process, it is reasonable to state that those pairs could be specific in pollen development. There are 5 clusters with more than one TF in each cluster. We search the coexpression for those TFs in each cluster, and find 9 pairs of TFs to present the relatively significant coexpression (in total 15 TFs; |r| >0.4). Almost all pairs of those coexpressed TFs are positively correlated, except one pair between At5g04760 and At5g43990 in cluster 3 (r = -0.41). In addition, we also search every pair of target genes in each cluster for the coexpression, and find 118 coexpression pairs with 6 highly correlated ones (r>0.8), which implies that the rest 112 pairs of coexpression genes in each cluster could be specific in the related stage of pollen development process.

### The regulatory dynamics of pollen development

**Figure 3:**
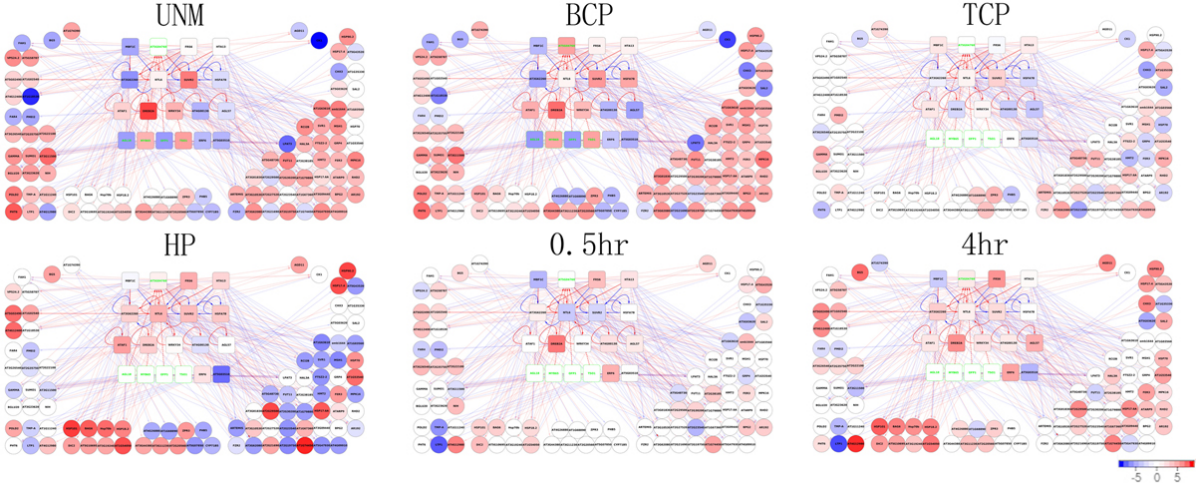
**A dynamic network of transcription during *A. thaliana *pollen development**. The pollen development of *A. thaliana *ranges from UNM, BCP, TCP, MP, HP, to 0.5 hr pollen tube, and 4 hour pollen tube stages. The transcription factors are represented as a square, and target genes as a circle. Blue or red arrow lines show the influence of a transcription factor on a target gene, positively or negatively. The transcription factors, that are not processed by NCA but collected by text-mining, include AGL18, OFP1, TSO1 and MYB65. The genes with no expression are denoted with green line. 11 clusters are grouped together in total.

According to the relationships inferred from NCA, we built an integrated model of *A. thaliana *pollen development (Figure 3). The final dynamic network integrates the inferred transcription factor activities, the regulatory relationships between TFs and their target genes, clustering on the adjusted strengths, the gene expression profiles, and the text-mining data. The network includes 19 TFs and 101 target genes. Several transcription factors present their specific dynamic expression pattern during the pollen development. For example, the expression of AT5G04760 is not detectable during UNM development stage, while AGL18, OFP1, TSO1 and MYB65 are not expressed during TCP, HP, 0.5 hr and 4 hr development stages. The rest genes present their expression during all of the pollen development processes and display different expression at least ones.

AT5G04760 is found no expression at UNM stage. From UNM to BCP stage, AT5G04760 is activated and interacts with SUVR2 to regulate their downstream gene expression. In contrast, AGL57 is deactivated during the stage switch. By the end of BCP stage, AT5G04760 and AGL57 have already executed their function and affected gene expression, including the genes in clusters 3, 4, 5, 10, and 11. From BCP to TCP stage, all genes show trends of not differently expressed. The pollen in TCP stage is similar to MP stage since the number of DEGs detected in both stages is very small. For transcription factors AGL18, OFP1, TSO1, and MYB65, they are curated to play the roles in pollen development from literature and therefore incorporated in the regulatory network. Those transcription factors show no detectable expression until into the 4 hr stage. Another transcription factor, DREB2A, is dramatically deactivated from the beginning. After TCP stage, DREB2A keeps steadily activated; until HP stage, it begins to restore to their basal level of activity. The temporal model therefore provides a global view of TFs' activation and the regulatory relationships between TFs and their target genes during the pollen development of *A. thaliana*.

**Figure 4:**
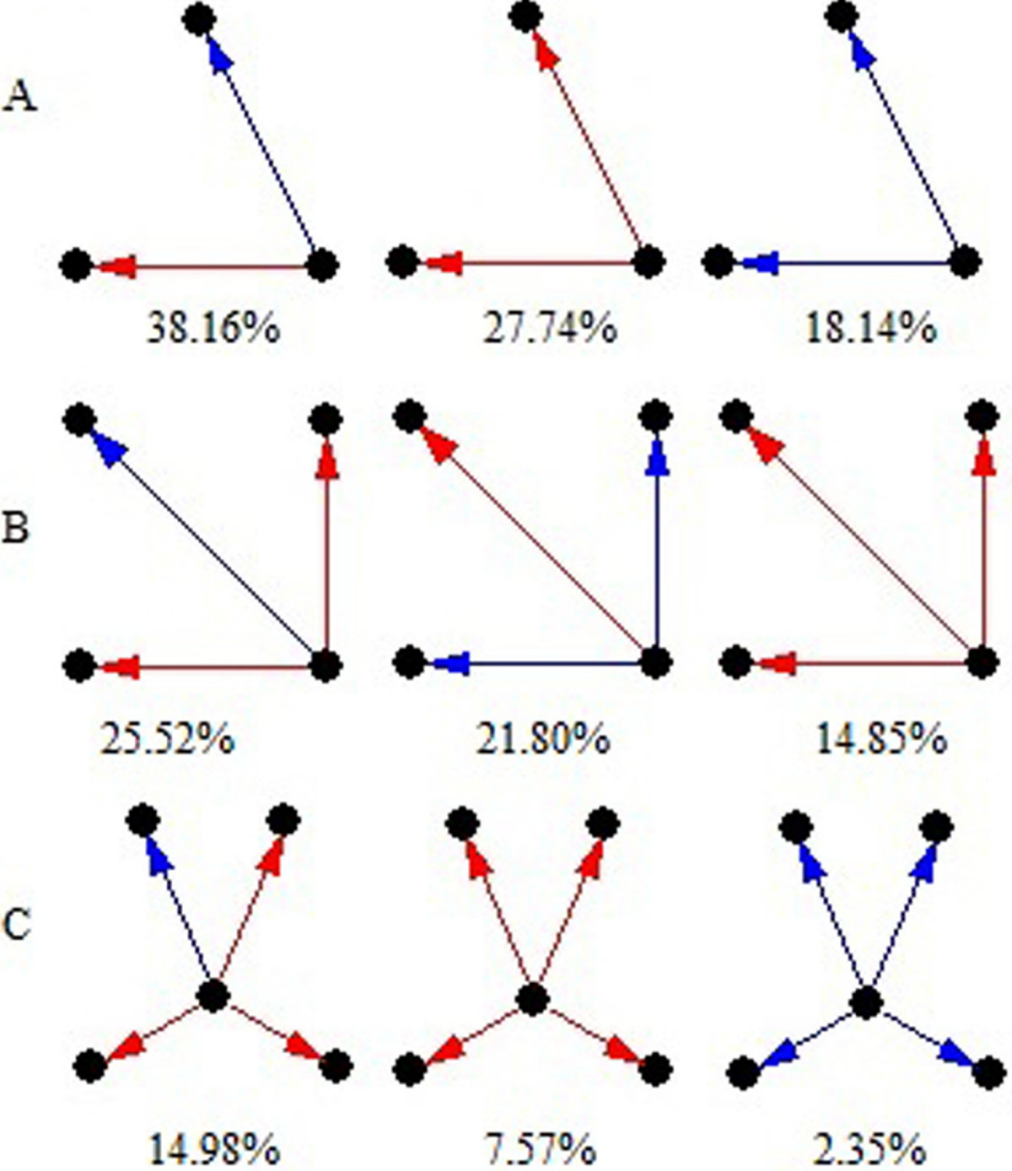
**The over-presented motifs**. A: Motif with size 3; B: Motif with size 4; C: Motif with size 5. Black nodes pointed to by an arrow are target genes, others are transcription factors. Red lines represent positive regulation, and blue lines negative regulation. The numbers represent the percentage of above motifs in the network.

The transcription networks have been proven to be made up of a small set of recurring regulation patterns that are called network motifs, and they serve as basic building blocks of transcription networks. To obtain the regulation pattern during pollen development, we detect network motifs in the network. In total, we retrieve 11 network motifs for motif size 3, 82 motifs with motif size 4, and 778 motifs with motif size 5. Each motif embodies a regulation pattern. And most all of the TFs display different roles in more than one regulation pattern. We detect the network motifs for all pollen development stage and find some interesting TF interactions (Figure 4).

For example, MBF1C, which expresses in pollen tube and enhances the tolerance to various biotic and abiotic stresses [38], displays the pattern of up-regulates AT3G62260 and down-regulates NTL6. AT3G62260 functions as protein serine/threonine phosphatase activity and NTL6 undergoes proteolytic processing. Our result indicates that MBF1C regulates protein serine/threonine phosphorylation and proteolysis in the opposite direction. Since phosphorylation plays an important role in the pollen-stigma interaction [39] and AT3G62260 is upregulated before TCP stage, it can be anticipated that MBF1C promotes the pollen-stigma recognition.

According to the network motif, WRKY34 upregulates other 3 target genes: FER3, RHD2 and GRP4 in the pollen development. FER3 has been reported to protect cells against oxidative damage [40] and RHD2 can lead the formation of reactive oxygen species [41], whereas overexpression of GRP4 can increase plant tolerance to osmotic stress [42].

Therefore, as a gene solely expressed in pollen, WRKY34 potentially promotes the expression of FER3, RHD2 and GRP4, which may function as a module to balance the reactive oxygen species metabolism during the process.

## Conclusions

The ultimate goal of our work is to construct a dynamic regulation network of pollen development. With NCA, we have predicted the activities of 15 transcription factors and the regulatory strengths of those TFs to their target genes. Based on the regulatory strength matrix, we have clustered the coexpressed and coregulated genes into different groups. By incorporating the regulatory network information with the regulatory strength matrix, we have further inferred the activities and interactions between transcription factors and their target genes.

The regulatory strength matrix is clustered to determine gene groups which are not only co-expressed, but also co-regulated. Identification of interactions between TFs and their target genes enable us to interpret the activation of regulatory relationship over development stage. Beyond the 15 TFs, we have also identified additional 4 TFs and explored the special expression pattern of the 4 TFs that are not included in the model, but are pollen development-related by text-mining. Moreover, WRKY34, which has been reported only expressed in pollen [43], has also been identified by NCA. We finally have reconstructed the dynamics of pollen development process of *A. thaliana *using above results. Moreover, we present the dynamic regulatory networks over all explored pollen development stages.

Although the NCA we used in this work can infer hidden TF activities by taking advantages of the prior of network structure, most of the regulatory information however is not available and the regulatory pairs retrieved from coexpression tend to be hypothetical. In addition, NCA is based on a phenomenal model of TFs' regulatory over target genes, which correlates with Hill cooperation between TFs, which do not potentially reflect the biological reality if we consider the complexity and multi-steps of the transcription event [44]. Nevertheless, in this study we combine all available datasets and construct a comprehensive dynamic network of the *A. thaliana *pollen development. This network characterizes the stage-specific activities of TFs of importance and the corresponding dynamics of this network during the stage of development. New relations between transcription factors and their target genes have been inferred from the network. Obviously, this network will shed new light on the study of mechanisms that governing the development of the pollen.

## Methods

### Data preprocessing

The gene expression datasets were obtained from Gene Expression Omnibus (GEO), with accession numbers: GSE6162, GSE6696, and GSE17343. The log2 ratio of genes expression in each development stage was calculated by MAS5 [45], with significance as p-value < 0.01. For all development stages we explored, the genes with at least differentially expressed at one stage were selected. In total, 5, 980 genes, which were differentially expressed (|FC|>1.6), were selected to be hierarchically clustered by hcluster of R language and to calculate the correlation coefficient for each pair of genes. For each pair of TF and its target gene, only the target gene in the sub-tree of the TF-node with the coefficient larger than 0.9 was kept for NCA.

### Network component analysis and dynamic network construction

Network component analysis (NCA) is a powerful mathematical tool for uncovering hidden regulatory signals from gene expression levels with a prior network structure information in terms of matrix decomposition [46]. The classical decomposition methods, such as PCA and ICA, assume orthogonality and independence, respectively, all of which lack biological foundation. On the other hand, the NCA does not make any assumptions on statistical properties and allows proper handling of prior network connectivity information.

NCA uses the standard log-linear model to approximate the relationship between levels of TFs activity and that of the target-gene expression by assuming the Hill cooperation between TFs on the promoter region of target genes. Formally:

(1) $$\frac{E_{i}\left(t\right)}{E_{i}\left(0\right)}=\overset{L}{\underset{j=1}{\prod }}{\left(\frac{TFA_{j}\left(t\right)}{TFA_{j}\left(0\right)}\right)}^{CS_{ij}}$$

Where t represents the time stage, *E_i_*(*t*) is the gene expression level and *TFA_j_*(*t*) is TF j's activities and *cs_ij _*reflects the control strength of TF j on gene i.

After logarithm, the equation (1) is linearized into (in forms of matrix):

(2) log[Er]=[CS]log[TFAr]

While the matrix [*Er*] consists of elements [*Er*]*_ij _*= *E_ij_*(*t*)/*E_ij_*(0) and similarly [*TFAr*]*_ij _*= *TFA_ij_*(*t*)/*TFA_ij_*(0), represents the relative gene expression levels and TFs' activities. The dimension of [*Er*] is *N *× *M *(N genes and M samples or conditions) while that of [*TFAr*] is *L *× *M *(L TFs). They respectively indicate the time courses of relative gene expression levels and TFs' activities. Finally, size of [*CS*] is *N *× *L*, which is the control strength for L TFs on each of N genes. The equation (2) above can be further simplified as:

(3) [E]=[S][A]

Here, we have the strength matrix, [*S*], which corresponding to the term of [*CS*] in equation (2) and the TFs' activity matrix [*A*], which is the equivalent of log[*TFAr*] in the equation (2), and finally, the gene expression matrix of [*E*] corresponds to the term of log[*Er*] in equation (2).

Based on above preparation, the decomposition of [*E*] into [*S*] and [*A*] can be achieved by minimizing the following object function:

(4) $$\begin{array}{c} \min||\left(\left[E\right]-\left[S\right]\left[A\right]\right)||\\ \text{Subject to}.\;S\in Z_{0} \end{array}$$

In NCA, the above target function is estimated by using the bootstrap algorithm and the value of [*S*] and [*A*] can be normalized through a nonsingular matrix of [*X*] according to,

(5) $$\left[E\right]=\left[S\right]\left[A\right]=\left[S\right]\left[X\right]\left[X^{-1}\right]\left[A\right]$$

Specifically, to guarantee uniqueness of the solution for the matrix decomposition of Eq. 4, the network topology needs to satisfy some criteria [9]: (i) The connectivity matrix [A] must have full-column rank. (ii) When a node in the regulatory layer is removed along with all of the output nodes connected to it, the resulting network must be characterized by a connectivity matrix that still has full-column rank. (iii) [P] must have full row rank.

The algorithm of NCA is already implemented in MATLAB by the authors, which is downloadable at http://www.seas.ucla.edu/~liaoj/. In this study, we followed the manual of this package and performed our computation.

With NCA, the significant TFs and their target genes were detected, the control strength of TFs to their target genes was recalculated, and the activities of the TFs were estimated. We took the control strength (only as positive or negative) as the regulatory relationships between TFs and their target genes (including TFs), and the TFs activities substitute for their gene expression to construct the dynamic network.

### Over-presented motifs among network

Motifs are small connected sub-networks that a network displays in significantly higher frequencies than would be expected for a random network. To uncover the regulation pattern of dynamic regulation network, we took FANMOD [47,48] to detect the over-presented motifs.

## Competing interests

The authors declare that they have no competing interests.

## Authors' contributions

Data collection: JGW. Programming: JGW. Design of the analysis process: TLS, JGW. Data analysis: JGW, TLS, YD, XJQ. Paper Writing: JGW, XJQ, YD, TLS and YHL. Paper Finalizing: TLS and YHL. All authors read and approved the final manuscript.

## Supplementary Material

Additional file 1 **Major clusters formed from the adjusted strength matrix and the target genes' GO functions**. Cluster of the genes. Click here for file
